# Molecular Alterations in Semen of Per-And Polyfluoroalkyl Substance Exposed Subjects: Association Between DNA Integrity, Antioxidant Capacity and Lipoperoxides

**DOI:** 10.3390/antiox14070792

**Published:** 2025-06-27

**Authors:** Carmela Marinaro, Anna Rita Bianchi, Valeria Guerretti, Gaia Barricelli, Bruno Berman, Francesco Bertola, Salvatore Micali, Francesco Paolo Busardò, Alessandro Di Giorgi, Anna De Maio, Marina Piscopo, Luigi Montano, Gennaro Lettieri

**Affiliations:** 1 Department of Biology, University of Naples Federico II, Via Cinthia, 21, 80126 Naples, Italy; carmela.marinaro@unina.it (C.M.); annarita.bianchi@unina.it (A.R.B.); valeria.guerretti@unina.it (V.G.); ga.barricelli@studenti.unina.it (G.B.); brunberm@libero.it (B.B.); gennaro.lettieri@unina.it (G.L.); 2 ISDE—Medici per l’Ambiente, Sezione di Vicenza, 36100 Vicenza, Italy; isde@isde.it; 3 Department of Urology, University of Modena and Reggio Emilia, 41121 Modena, Italy; salvatore.micali@unimore.it; 4 Department of Biomedical Sciences and Public Health, Marche Polytechnic University, 60020 Ancona, Italy; f.busardo@univpm.it (F.P.B.); s1108252@pm.univpm.it (A.D.G.); 5 Andrology Unit and Service of LifeStyle Medicine in Uro-Andrology, Local Health Authority (ASL) Salerno, 84124 Salerno, Italy; l.montano@aslsalerno.it

**Keywords:** human sperm, antioxidant activity, lipoperoxide, PFAS, DNA damage, pollution, Veneto Region

## Abstract

In the last decades, there has been huge interest in Per- and Polyfluoroalkyl Substances (PFAS) worldwide because of the toxic effects on humans. In 2013, a large-scale contamination of PFASs in the Veneto region was caused by a fluorochemical plant in Vicenza. About 130,000 inhabitants were exposed to PFAS in their drinking water. To date, relatively few studies have investigated the associations between blood serum PFAS concentrations and oxidative stress in semen. This study compared the antioxidant activity, lipoperoxide levels and protection or induction of oxidative DNA damage by sperm nuclear basic proteins (SNBP) in subjects living in Veneto exposed to PFAS (VNT) with those living in a non-PFAS contaminated area (VSL). Although the semen parameters were within the WHO range, the VNT semen samples showed higher levels of lipoperoxides and lower antioxidant activity compared to the VSL samples. These differences were statistically significant. We also examined DNA damage following SNBP addition under pro-oxidative conditions, finding a significantly different distribution of DNA damage types between the two groups, where 0 means no damage and 1 to 3 means increasing damage with 3 indicating maximum damage. SNBP of VNT subjects showed a reduced ability to protect DNA from oxidative damage. In the VSL group, damage 0 was found in 56% of subjects, 35% of the VNT group show damage 1, 36% damage 2 and 18% damage 3, while only 11% of VNT subjects show damage 0. Additionally, VNT with 0-grade DNA oxidative damage also exhibited reduced antioxidant activity and higher levels of lipoperoxides, in contrast to VSL. The results of this study indicate that exposure to PFAS produces oxidative stress in the semen of VNT subjects, who were also found to have blood serum perfluorooctanoic acid (PFOA) levels above the threshold. This suggests the possibility of infertility issues and emphasises the necessity for additional research into the long-term consequences of oxidative stress on male fertility and the health of offspring.

## 1. Introduction

Male infertility remains a clinical challenge with several contributing factors, among which lifestyle and environmental exposures seem to be very important in recent years [1,2,3,4]. Molecular studies of the causes leading to male infertility have shown a considerable role for oxidative stress (OS), which occurs when excessive amounts of reactive oxygen species (ROS) are generated or antioxidant activity is compromised, resulting in an imbalance of oxidation and reduction [5,6,7]. Sperm are particularly sensitive to ROS. They possess extremely low concentrations of enzymatic antioxidants, insufficient to provide protection against high ROS levels, and the cytoplasm has low amounts of the enzyme that can neutralize them [8,9,10,11,12,13]. Damage to spermatozoa mediated by ROS has a major impact on male idiopathic infertility [14,15,16,17]. ROS can have a beneficial or harmful effect on sperm depending on the concentration, location and time of exposure. At a physiological level, ROS are indispensable for sperm motility, induction of sperm capacitation, acrosome reaction, hyperactivation and hence fertilization [18,19,20,21,22]. Excessive production of free radicals beyond the antioxidant capacity of sperm and seminal plasma can result in lipid peroxidation, apoptosis, protein and DNA damage and low sperm quality [23,24,25,26]. ROS are also implicated in the condensation of sperm chromatin, which regulates the abundance of reproductive cells by directing the apoptosis or proliferation of spermatogonia [27]. The integrity of sperm DNA has been shown to be permanently compromised in the presence of increased susceptibility to OS [28,29,30], and a number of studies have demonstrated the vital role of intact sperm DNA in the development of normal embryos [31,32,33,34,35,36] and pregnancy rates [37]. Over the last 50 years, development and industrialisation have led to chemicals being released into the environment, causing irreversible damage to human health [38]. Per- and Polyfluoroalkyl substances (PFAS) are harmful chemicals that accumulate and persist in the environment. They have been used in products for almost 60 years due to their stability, but have led to widespread contamination. They have been detected in various biological systems, but most significantly in groundwater used for drinking, which is harmful to living organisms [39,40]. Drinking contaminated water over time can lead to these substances building up in the body, taking years to decrease. PFAS are also “endocrine-interfering agents” (EIs) [41,42,43,44] and it has been amply demonstrated that EIs have the greatest impact on the reproductive phase of life, from gamete production to fertilisation and offspring development. Since the discovery of PFAS contamination in drinking water worldwide, scientists have been studying its health effects. These studies have shown that PFAS can cause oxidative stress and that exposure to PFAS is associated with infertility and other diseases [45,46,47,48]. In spring 2013, it was found that groundwater in Veneto was contaminated with PFAS from a plant that had been operating since the late 1960s. A study by the National Research Council (CNR) and the Ministry of the Environment revealed significant contamination of groundwater, surface water, and drinking water in the provinces of Vicenza, Padova and Verona. Residents in the Veneto region were exposed to high levels of these substances, particularly perfluorooctanoic acid (PFOA) [49], in their drinking water until autumn 2013. The Veneto Regional Agency for Environmental Prevention and Protection (ARPAV) investigated the contamination of groundwater and drinking water in the region. Twelve types of PFAS were identified, and the source was traced back to a PFAS production plant in Trissino.

**Figure 1:**
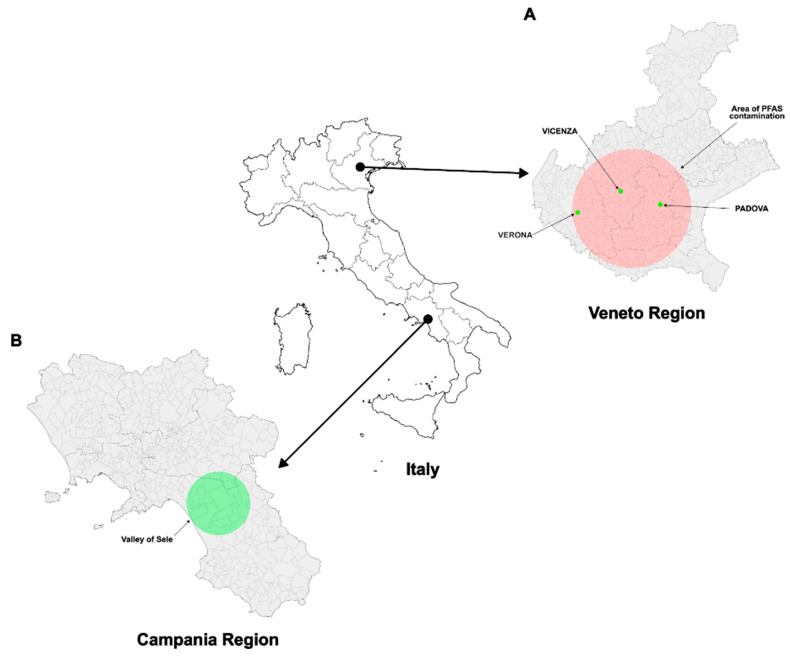
Map of recruitment areas. (**A**) Veneto Region; (**B**) Campania Region.

Contamination of the groundwater led to exposure of 130,000 people to these compounds. In 1980, ARPA (Regional Environmental Protection Agency) estimated that groundwater contamination had reached the public aqueducts serving municipalities in the provinces of Vicenza, Verona and Padova. A 2016 study found that people living in areas with high levels of PFAS had higher serum levels of PFAS than those living in areas with low levels [50]. Women also had lower concentrations of PFOA and perfluorooctanesulfonic acid (PFOS) than men, which appears to affect reproductive health [51,52]. Pollution has been found to negatively impact sperm quality, resulting in changes to standard sperm parameters. It has also been linked to DNA fragmentation and the modification of properties of certain proteins. This includes the modification of sperm nuclear basic proteins (SNBPs) in young men living in polluted areas of Italy [53], and in some species of marine life [54,55,56]. During sperm production, protamines replace histones and compact the chromatin of the sperm. Pollution also affects protamination. An imbalance can make DNA more vulnerable to oxidative stress [57]. In the present study, lipoperoxide levels and antioxidant capacity were measured for the first time in seminal plasma in subjects living in the Veneto region (VNT) (provinces of Vicenza, Padova and Verona) compared to subjects living in an area not exposed to PFAS contamination (the Sele Valley in the Campania region, VSL). This is a low environmental pressure area, as reported in Pizzolante et al. 2021 [58]. In addition, the ability of SNBPs to protect and induce oxidative DNA damage in VNT and VSL groups was also analysed in order to use this parameter as a potential indicator of sperm health. For this purpose, semen samples were analysed from 83 male subjects aged 18–35 (born after 1985), who had lived in one of the municipalities in the VNT for at least five years between 1985 and 2017, or whose mothers were pregnant or breastfeeding in the same period. The control group consisted of semen samples from 50 subjects of the same age living in VSL (Figure 1).

## 2. Materials and Methods

### 2.1. Ethical Statements

The study was performed in accordance with the guidelines and regulations described by the Code of Ethics of the World Medical Association (Declaration of Helsinki) and falls within the scope of the EcoFoodFertility project (https://www.ecofoodfertility.it; accessed on 23 June 2025). The study protocol was approved by the Ethics Committee for Clinical Trials of the province of Vicenza on 8 November 2019 (prot. 113421) and, for some changes made, approved again on 29 July 2021 (prot. 79483).

### 2.2. Recruitment

This cross-sectional study included a planned preliminary evaluation of a data subset. This analysis was designed to yield broader results earlier in the study, rather than waiting for all analytical determinations from the entire sample. Data from 507 young adults (18–35 years old) enrolled between 2022 and 2023 and living in municipalities in the so-called red area (provinces of Padova, Verona and Vicenza) were used in this preliminary analysis. In particular, DGR 2133/2016 and 691/2018 of the Veneto Region defined the municipalities supplied with polluted drinking water. It was decided to carry out the registration only in the communes involved in the supply of polluted drinking water for the whole territory of the commune. For the present study, these communes were as follows: Albaredo d’Adige, Alonte, Arcole, Asigliano, Bevilacqua, Boschi S. Anna, Brendola, Cologna Veneta, Legnago, Lonigo, Minerbe, Montagnana, Noventa Vicentina, Orgiano, Pojana Maggiore, Pressana, Roveredo, Sarego, Terrazzo, Urbana, Veronella and Zimella. Specifically, this study analyzed the semen of 83 men, aged 18–35 (born after 1985) who had lived in one of the municipalities in the provinces of Vicenza, Padova and Verona (VNT) (Figure 1), for at least 5 years between 1985 and 2017, or whose mothers had been pregnant or breastfeeding in the same area during the same period. The semen of these subjects was compared to that of 50 men of the same age from the Sele Valley (VSL), used as a control area. This latter was a low environmental impact area (https://www.arpacampania.it/; accessed on 23 June 2025) with no known illegal dumping of toxic waste and had an economy based mainly on low to medium scale agriculture. On the basis of the pollutant concentrations detected in the area, the Sele Valley was in the “maintenance” category [59].

#### 2.2.1. Inclusion Criteria

The following are the criteria for inclusion in the study:

1. The first valid birth date for enlistment was the year 1985, because ARPAV assessed in the early 1980s the years when the contaminated river of groundwater from Trissino reached the Almisano well field, from where deep water was captured and distributed to the 23 exposed municipalities.
2. The participant must have been born or have lived for at least 5 years in one of the 23 municipalities that the Veneto region has defined in its resolution as a “red zone”, because PFAS pollution of deep, surface and drinking water has been demonstrated in this area.
3. Spontaneous and voluntary enrolment was achieved through registration and reservation by the participant of the day chosen for visits on an appropriate digital platform.
4. Each enrollee was sent a digital questionnaire containing over 100 questions to explore the residential history of the subject and their mother during pregnancy and breastfeeding. Completion of the questionnaire was a prerequisite for participation in subsequent phases of the study.

#### 2.2.2. Exclusion Criteria

All subjects with at least one of the following criteria were excluded from sampling:

1. Born and residing in an area other than the red zone, under the age of 18 or over the age of 35.
2. Genetic factors (all genetic syndromes, i.e., Klinefelter, Down, etc.)

Each participant’s compliance with the inclusion and exclusion criteria was checked both through the questionnaire and through participant interviews conducted by ISDE (International Society Doctors for the Environment) prior to the start of the clinical-instrumental analyses.

#### 2.2.3. Confounding Factors

The following clinical and pharmacological situations were evaluated as covariables. These were ascertained by both testicular ultrasound and a specific urological examination, which were performed on all study participants:

1. Varicocele; orchitis, urethritis, prostatitis, epididymitis of infectious and non-infectious nature.
2. Presence of severe systemic diseases and related therapeutic treatments known to procure worsening of fertility, such as type II diabetes; intercurrent infectious diseases; neoplastic diseases, taking drugs that interfere with spermatogenesis (e.g., antidepressants, anxiolytics, antibiotics, corticosteroids, etc.); sex hormone therapies; and performing cycles of chemo/radiation therapy.

### 2.3. Determination of Spermiograms

Semen samples analysis was carried out at the SE.FA.MO (Vicenza, Italy) outpatient clinic in Vicenza. Semen samples were collected and immediately analysed according to the WHO 2021 guidelines [60]. The sperm parameters analysed were the following: semen volume (mL), total number of sperm cells (1 × 10^6^), sperm viability (%), normal sperm cells (%), total sperm motility (%) and progressive sperm motility (%).

### 2.4. Anti-OX Sperm Test

Total antioxidant capacity (TAC) was determined using a commercial test kit, Anti-OX Sperm (Diacron International, Grosseto, Italy), on seminal plasma of the VSL group and the VNT group exposed to PFAS contamination in drinking water. This method exploited the ability of the antioxidants present in the sample to reduce ferric iron (Fe^3+^) to ferrous iron (Fe^2+^), which reacted with a chromogen to produce a reddish-purple color, the intensity of which increased in proportion to the amount of iron reduced by the antioxidants present in the sample. Measurements were made of exogenous non-enzymatic antioxidants (vitamin C, vitamin E and polyphenols) and endogenous non-enzymatic antioxidants (thiol groups (SH), uric acid and albumin) present in the sample. The procedure consisted of incubating a mixture of 1 mL of reagent 1 (chromogen) and 10 µL of sample at 37 °C for 5 min in the dark after gentle agitation. Spectrophotometric measurements were performed at 505 nm. A standard sample of reduced iron (1000 uEq/L) and a blank, in which plasma was replaced by an equal volume of water, were also subjected to the same procedure. Total antioxidant capacity (TAC) was expressed as µmol/L Fe^3+^ reducing antioxidants per liter of sample.

### 2.5. Lipoperoxides (LP) Sperm Test

The determination of LP was performed on seminal plasma samples of the VSL group and VNT group exposed to PFAS contamination in drinking water. The analyses were performed by the LP Sperm Test Kit (Diacron International, Grosseto, Italy) [61]. The test was based on the ability of peroxides to promote the oxidation of Fe^2+^ to Fe^3+^. The peroxidation product (Fe^3+^) bound to the chromogen, forming a colored complex, which was measured spectrophotometrically at 505 nm. The increase in absorbance was directly proportional to the concentration of LP in the sample. The procedure started with the addition of 1 mL of reagent 1 supplied with the kit and 10 μL of sample. After adding three drops of reagent 2 (chromogen) and 10 μL of reagent 3, the mixture was vortexed and incubated at 37 °C for 5 min. The mixture was then centrifuged at 1400× *g* for 2 min and the supernatant was used for absorbance measurement. A standard sample consisting of ferric chloride solution (1000 uEq/L) and a blank, in which the plasma is replaced by an equal amount of ddH_2_O, was analysed according to the procedure described above. The concentration of LP was expressed as µmol/L.

### 2.6. Plasmid pGEX-2TK Extraction

A pGEX-2TK plasmid (4969 bp) from *E. coli* HB 101 cells was extracted by using the ZymoPURE™ Plasmid Midiprep Kit (Zymo Research Europe, Breisgau, Germany). The extraction was performed following the manufacturer’s instructions, with little modification regarding the temperature used as described in Carbone et al. 2012 [62]. The plasmid was quantified by NanoDrop (Thermofisher, Waltham, MA, USA) and run on 1% agarose gels to determine the state.

### 2.7. Extraction of Sperm Nuclear Basic Protein (SNBP) from Spermatozoa

Spermatozoa were isolated from seminal plasma through centrifugation at 5500× *g*, 30 min at 4 °C. SNBP extraction followed a modified protocol based on previous studies [63]. Sperm pellets were washed twice with 500 μL PMSF, followed by resuspension in 50 μL of 1 mM PMSF combined with 50 μL of 6 M guanidinium chloride/10 mM DTT solution and then incubated at 20 °C for 30 min. Chromatin precipitation was achieved by adding 5 volumes of cold absolute ethanol with subsequent incubation at −20 °C, minimum of 60 min. After centrifugation 13,680× *g* for 15 min at 4 °C, the pellet was resuspended in 500 μL of 0.5 M HCl, incubated at 37 °C for 5 min and centrifuged 1000× *g* for 10 min at 4 °C. SNBPs were precipitated from the supernatant using 20% trichloroacetic acid (TCA) and incubated at 4 °C for 60 min, followed by centrifugation 14,000× *g* for 10 min at 4 °C. The resulting pellet was washed with 500 μL of acetone containing 1% β-mercaptoethanol and twice centrifuged at 14,000× *g* for 10 min at 4 °C. The pellet was dried by speed-vacuum (10–15 min). The extracted proteins were then resuspended in 50 μL of ultrapure water (milliQ) (Millipore Merck, Burlington, MA, USA) and either utilized immediately or stored in 50 μg aliquots at −20 °C.

### 2.8. DNA Protection Analysis

The ability of SNBP extracted from VSL and VNT groups, to protect DNA from oxidative damage, in the presence of 12 µM H_2_O_2_ and 10 µM CuCl_2_, a condition in which the Fenton reaction occurs, was assessed as described in Lettieri et al., 2021 [64]. In brief, to the fixed amount of plasmid DNA (pGEX-2TK), 150 ng, an increasing amount of SNBP was added. Protein/DNA weight/weight (*w*/*w)* ratios ranging from 0.4 to 0.8 were used. After 5 min of interaction of protein and DNA at RT, the H_2_O_2_ and CuCl_2_ were added and incubated at 37 °C for 30 min to induce oxidative damage. The result was analysed on 1% agarose gel at 100 V for 30 min in TEB 1X and stained with SafeViewTM classic (abm, Richmond, BC, Canada). Gel images were purchased from GelDoc Biorad (Hercules, CA, USA).

In particular, we have purposefully created a damage condition for plasmid under these conditions. This purpose-made damage condition is shown in well 3 of all agarose gels reported in Supplementary Figure S1. In this experiment, oxidative DNA damage was detected by the conversion of a fraction of the supercoiled plasmid DNA into relaxed. SNBPs were used to test their ability to aggregate and protect DNA from oxidative damage or induce it. The extent of DNA damage was graded as damage 0 (no damage), 1, 2 and 3 (maximum damage) as shown in the Supplementary Figure S1. We referred to the condition as grade 0 oxidative DNA damage (Supplementary Figure S1A) when the addition of SNBPs in the ratios 0.4 to 0.8 to DNA, produced the gradual disappearance of the supercoiled DNA that was found in the proximity of the well and thus complexed with SNBPs, which was indicative that these SNBPs had the capacity to aggregate DNA, protecting it from oxidative damage. In fact, no damage (i.e., an increase in the intensity of the relaxed plasmid DNA band compared to the condition shown in well 3 of Supplementary Figure S1) was observed with the addition of SNBPs to DNA.

Supplementary Figure S1B shows the oxidative DNA damage that we defined as grade 1. In this condition, the addition of SNBPs in ratios 0.4 to 0.8 to DNA (wells 4, 5 and 6) produced a slight increase in the intensity of the relaxed DNA band, but only in the 0.4 SNBP/DNA ratio and a decrease in the 0.6 and 0.8 ratios, compared to the condition shown in well 3. In addition, it is observed that a fraction of the supercoiled DNA is close to the well and thus this indicates that the SNBPs of these subjects had a fair ability to aggregate DNA, producing only a slight increase in DNA damage solely at the 0.4 protein/DNA ratio.

In the representative grade 2 damage, shown in Supplementary Figure S1C, compared to the damage condition we created (well 3) there was an increase in the intensity of the band corresponding to relaxed DNA following the addition of SNBPs in ratios of 0.4 to 0.8 (wells 4, 5, 6) to DNA. In addition, a high fraction of supercoiled DNA remained, indicating that the SNBPs of these subjects had a low capacity to aggregate DNA and the absence of protein-aggregated DNA in the proximity of the well. Finally, the grade 3 damage shown in Supplementary Figure S1D implied that the addition of SNBPs caused almost all the DNA to become relaxed, as shown by the increase in intensity of the band corresponding to the relaxed DNA. This suggested that SNBPs from these individuals had a very low capacity to aggregate DNA and consequently a poor ability to protect DNA from oxidative damage.

### 2.9. Determination of PFAS

An aliquot of 200 µL was fortified with internal standard and 600 µL of acetonitrile was added for protein precipitation. The supernatant was collected and transferred into a polypropylene tube containing 200 mg of QuEChERS salts (MgSO_4_:NaCl 4:1, *w*/*w*). The samples were vortexed and centrifuged at 4000 rpm for 10 min. The supernatant was collected, transferred to a new polypropylene tube and evaporated under nitrogen flow. Samples were reconstituted in 100 µL of water:methanol 80:20 (*v*/*v*), transferred into polypropylene autosampler vials, prior to injection of 10 µL into UPLC-MS/MS.

The separation of the analytes was performed using an ACQUITY UPLC BEH C18 column (2.1 mm × 100 mm, 1.7 µm, Waters Corporation, Milford, MA, USA). The mobile phases consisted of 2 mM ammonium acetate in water:methanol 95:5 (mobile phase A) and 2 mM ammonium acetate in methanol (mobile phase B), using an elution gradient. The column temperature was set at 35 °C. The mass spectrometer was equipped with an electrospray source in negative mode (ESI-) and was set in MRM (multiple reaction monitoring) mode on two transitions for each analyte, where possible.

### 2.10. Statistical Analysis

Statistical analyses were performed by the Mann–Whitney test, comparing the levels of LP and antioxidant capacity between the VSL and VNT groups. Regarding the comparison between the LP levels in the VSL damage type 0 group and the LP levels in the damage subgroups in the VNT group, the data were previously transformed to comply with the normality distribution, and the one-way ANOVA test was applied. A classic one-way ANOVA test was first performed. This analysis showed that the standard deviation between the groups was significantly different (Brown–Forsythe test, *p*-value: <0.0001; and Bartlett’s test, *p*-value: <0.0001). Therefore, Welch’s ANOVA test was chosen. The adjusted *p*-values of multiple comparisons of Welch’s test are shown in Comparison between the LP levels in the VSL damage type 0 group and the LP levels in the damage subgroups in the VNT group. The same statistical tests were applied for the comparison between the antioxidant capacity levels in the VSL damage type 0 group and the LP levels in the damage subgroups in the VNT group.

Also shown in Figure 5 are the adjusted *p*-values of Welch’s test. n = 3 is the number of technical replicates performed for each sample analysed

Statistical analyses were performed using GraphPad Prism 10.4.2 (Boston, MA, USA).

## 3. Results

### 3.1. LP and Antioxidant Capacity

**Figure 2:**
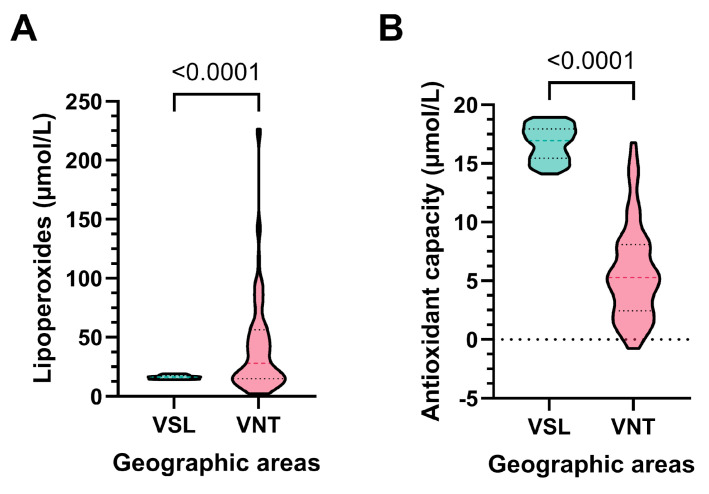
Violin plot of LP (**A**) and antioxidant capacity (**B**) evaluated in seminal plasma of VSL and VNT groups.

Figure 2 shows the levels of LP and antioxidant capacity measured in the seminal plasma of the VSL and VNT groups. VNT group presented significantly higher levels of LP than those measured in VSL (*p* < 0.0001) (Figure 2A). The VNT group also showed significantly lower levels of antioxidant capacity (Figure 2B).

### 3.2. DNA Oxidative Damage Protection Assay

**Figure 3:**
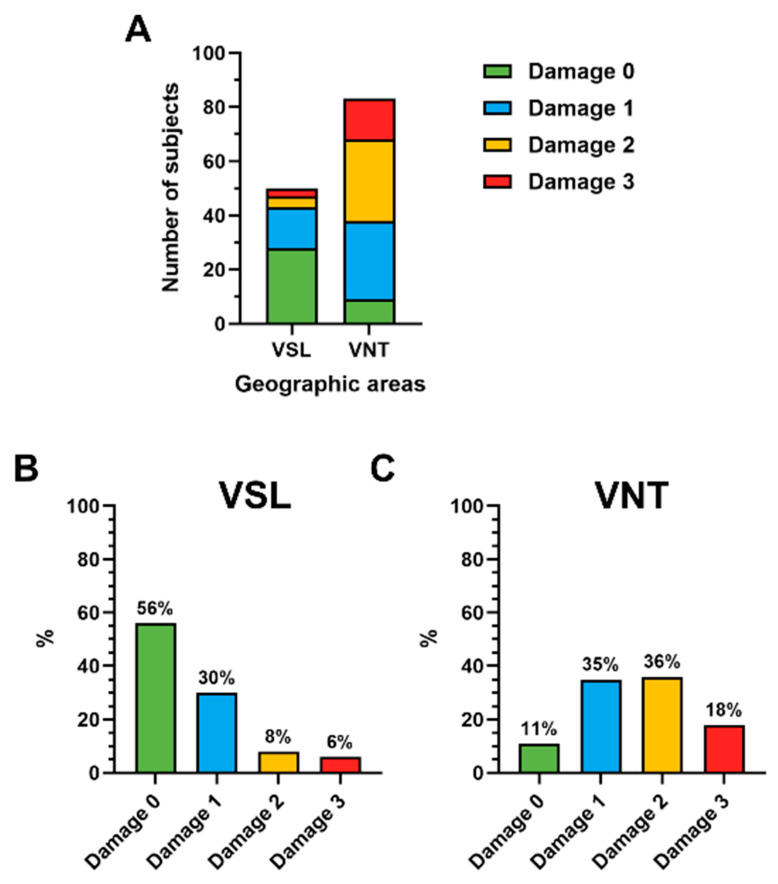
Stacked histogram showing the four types of oxidative DNA damage found in the VSL and VNT groups (**A**); percentage distribution in the two groups, VSL (**B**) and VNT (**C**), of the various types of damage.

Under pro-oxidative conditions, SNBP of VNT subjects showed a reduced ability to protect DNA from oxidative damage. In contrast to the VSL group, where damage 0 was found in 56% of subjects, 35% of the VNT group showed damage 1, 36% damage 2 and 18% damage 3 (Figure 3), while only 11% of VNT subjects showed damage 0 (Figure 3).

**Figure 4:**
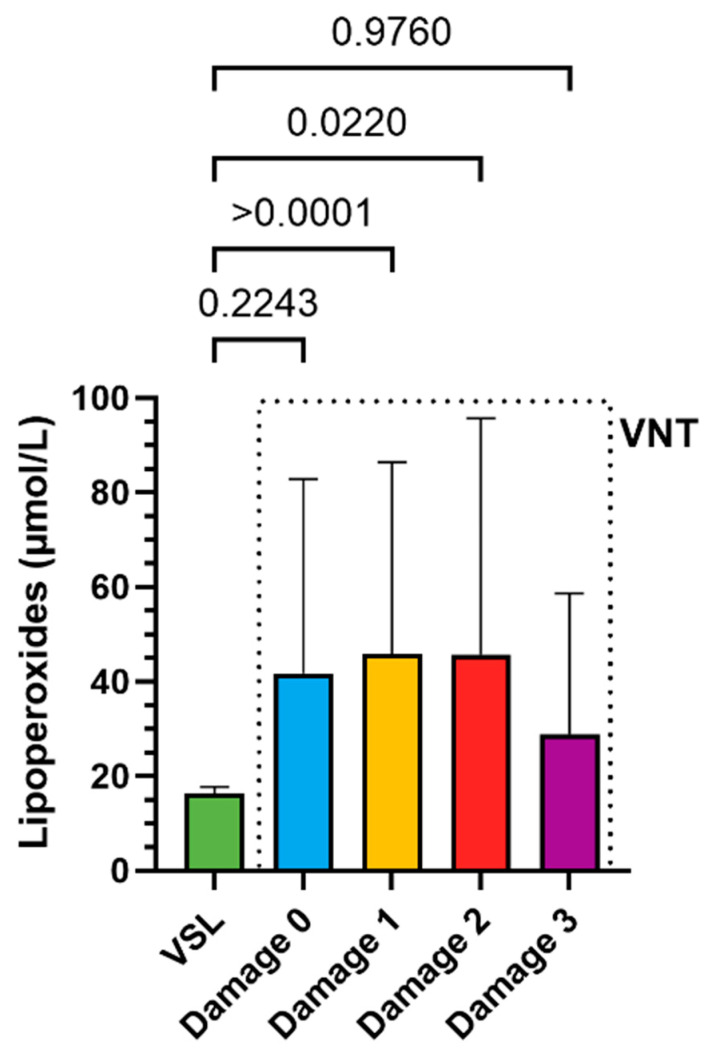
Comparison between the LP levels in the VSL damage type 0 group and the LP levels in the damage subgroups in the VNT group. Comparisons were performed using Welch’s ANOVA test (n = 3).

**Figure 5:**
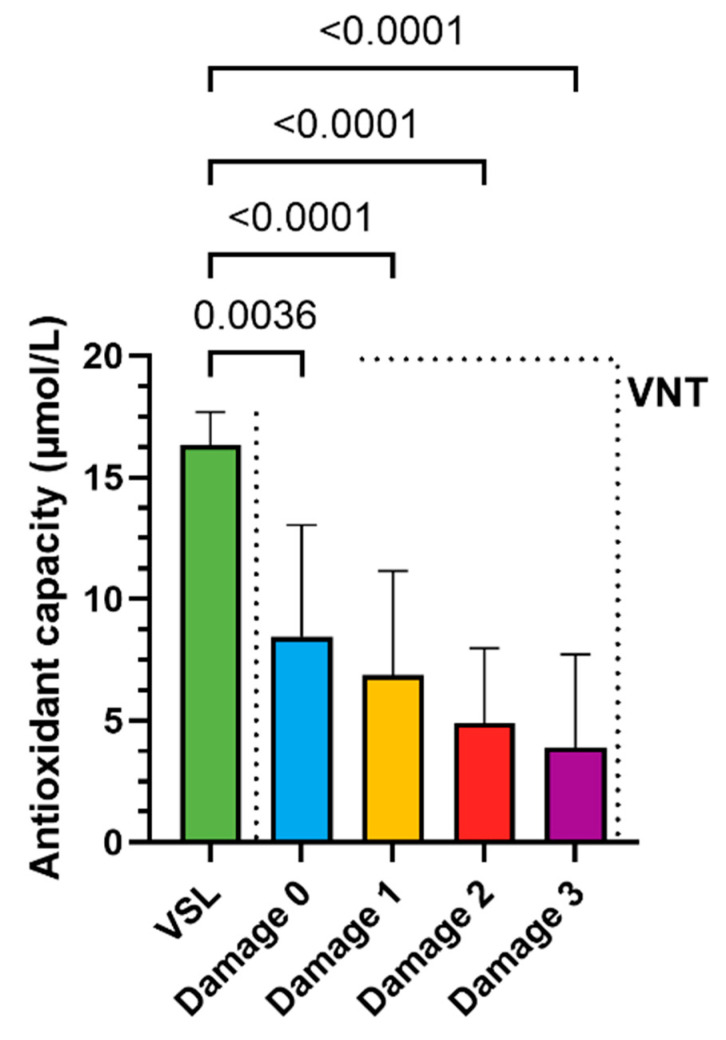
Comparison between the antioxidant capacity levels in the VSL damage type 0 group and the LP levels in the damage subgroups in the VNT group. Comparisons were performed using the Welch’s ANOVA tests (n = 3).

The levels of LP and the antioxidant capacity of the VNT group were also distributed according to different levels of DNA damage. The 0 damage VSL group was considered as control. PFAS contamination caused an increase in LP concentration (Figure 4) and a decrease in antioxidant capacity (Figure 5) already in the VNT group with 0 damage.

### 3.3. PFAS Determination

**Figure 6:**
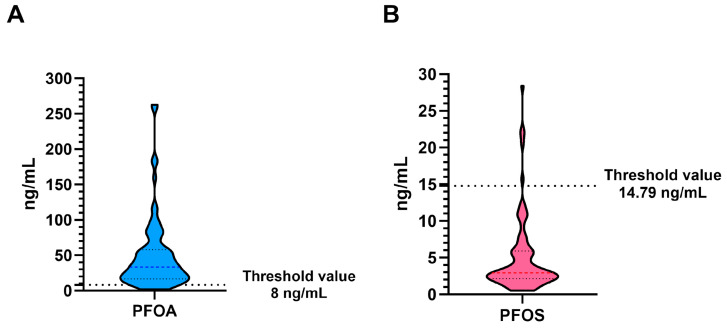
Violin plot of PFOA (**A**) and PFOS (**B**) in VNT group.

Figure 6 shows the levels of PFOA and PFOS in the blood serum of VNT subjects. The results showed that the PFOA levels were all above the threshold (Figure 6A), while the PFOS levels were in the normal range (Figure 6B). Serum concentrations of 8 ng/mL for PFOA and 14.79 ng/mL for PFOS were proposed as threshold values and denote a patient in good health, as reported in the health surveillance plan for the population exposed to PFAS [65].

### 3.4. Spermiograms

**Figure 7:**
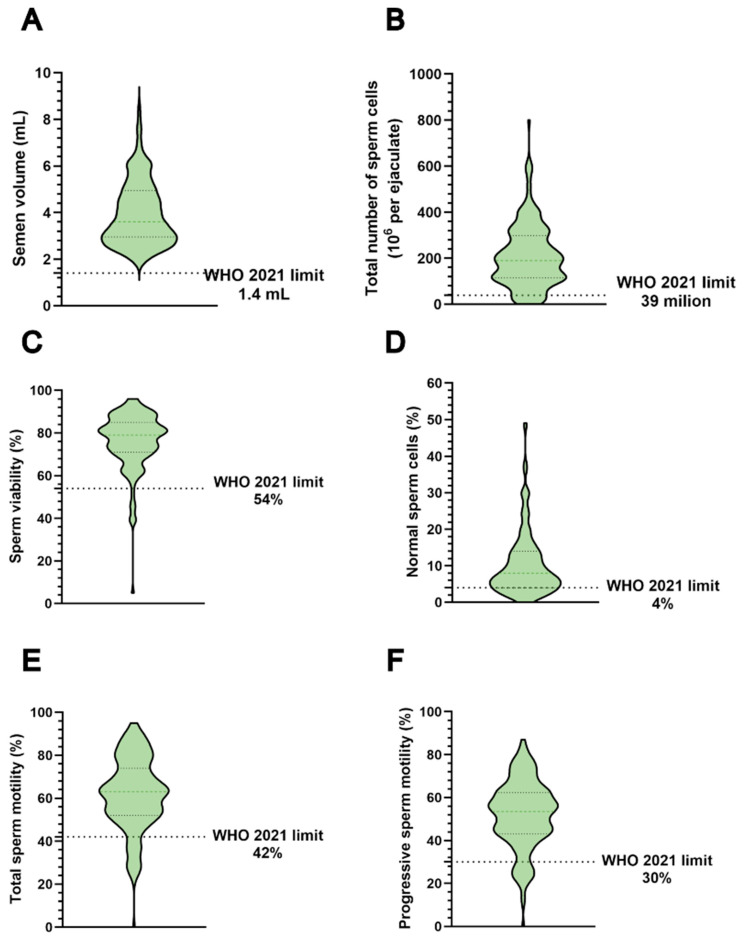
Spermiograms of VNT group: semen volume (**A**), total number of sperm cells (**B**), sperm viability (**C**), normal sperm cells (**D**), total sperm motility (**E**) and progressive sperm motility (**F**).

The parameters analysed in the spermiogram were semen volume (A), total number of sperm cells (B), sperm viability (C), normal sperm cells (D), total sperm motility (E) and progressive sperm motility (F). The results are shown in Figure 7, and the analysis revealed that most of the parameters were above the limit set by the WHO in 2021.

## 4. Discussion

Currently, male infertility is a major global public health problem affecting about 15% of couples of reproductive age worldwide [66,67,68], and environmental pollution has been shown to affect sperm quality [69,70,71,72]. Information concerning the effects of PFAS on sperm quality is still controversial [73,74,75,76,77,78]. This study was carried out as part of the EcoFoodFertility project and aimed to verify the effect of PFAS contamination on male fertility by comparing VNT and VSL subjects. Evidence of higher seminal lipid peroxidation (LP) levels and reduced antioxidant capacity in the VNT group compared to the VSL group suggests that PFAS exposure promotes sperm membrane lipid peroxidation and reduces reactive oxygen species (ROS)-neutralising antioxidant defences. Lipoperoxidation represents a critical mechanism of cellular damage in human spermatozoa exposed to oxidative stress [79,80]. The high polyunsaturated fatty acid content of human sperm membranes makes them particularly susceptible to oxidative damage [81,82,83,84]. This process produces cytotoxic aldehydes such as 4-hydroxynonenal (4HNE) and acrolein (ACR), which research shows significantly impair sperm function through multiple mechanisms: altered membrane fluidity affecting motility [81,85,86], mitochondrial dysfunction affecting energy production, creation of self-perpetuating oxidative cycles, DNA fragmentation [87,88] and activation of apoptotic pathways leading to cell death [89,90].

It was particularly concerning that compromised antioxidant capacity and increased LP were already demonstrated by the VNT group with grade 0 DNA damage compared to their VSL group, indicating that detectable DNA damage is preceded by biochemical alterations. Therefore, these analyses can predict an impaired oxidative state before more serious molecular changes occur that could affect male fertility. Studies showing negative correlations between lipid peroxidation markers (TBARS) and sperm concentration/count support the critical relationship between oxidative balance and sperm quality [1,91]. Antioxidant defence systems, such as superoxide dismutase (SOD) and glutathione peroxidase 4 (GPx4) [92], have been shown to have a protective effect. GPx4 is specifically linked to better sperm motility, and SOD is linked to reduced protein oxidation [93,94,95]. Moreover, previous studies have shown that other pollutants, including heavy metals and VOCs, alter sperm protamine/histone ratios and implicate SNBP in oxidative DNA damage [96,97]. There is a growing body of evidence suggesting a negative association between exposure to PFAS and semen quality [74,98,99]. However, the mechanisms by which PFAS may directly impact sperm biology are still unclear.

We have begun to assess the levels of PFOA and PFOS found in the blood serum of VNT subjects. The results revealed elevated PFOA levels and normal PFOS levels. A good health threshold was set at 8 ng/mL for PFOA and 14.79 ng/mL for PFOS. This is according to the health surveillance plan for the population exposed to PFAS [65]. In particular, the new European Drinking Water Directive (Directive (EU) 2020/2184), which Member States, including Italy with Legislative Decree 18/2023, set a threshold for PFAS intake of 0.50 µg/L. The directive will come into force by 12 January 2026. The previous drinking water directive, known as Directive 98/83/EC, did not set specific and binding European limits for PFOS, PFOA or other PFAS family compounds.

Regarding the DNA oxidative damage protection assay, the results showed that SNBP from VNT was ineffective in protecting DNA from oxidative stress for most of the samples, causing predominantly type 3 DNA damage. The behaviour of SNBP could be changed by the very high levels of PFOA. It is possible to hypothesise a link between SNBP and PFOA that induces conformational changes in these proteins, affecting their binding to DNA. An alternative hypothesis to be considered is that PFOAs have the capacity to induce post-translational changes in the SNBP by means of altering the manner in which they bind to the DNA.

Bearing in mind that semen accumulates pollutants more than other matrices [100], analyses to determine the presence of PFAS in the semen of these individuals can only provide a more accurate and detailed study of the effects of these substances on SNBPs, as has been demonstrated for heavy metals in *Mytilus galloprovincialis* and humans [101,102,103,104,105]. When we analysed the spermiogram parameters of the VNT subjects, we saw that for most of the parameters, the limits set by the WHO in 2021 were exceeded. Therefore, the analysed samples do not show any seminal changes. This is in line with results derived from in vitro exposed mouse spermatozoa to a relevant mixture of PFAS molecules [106]. Despite the acute sensitivity of the sperm cell, these authors showed that short-term in vitro PFAS exposure did not affect sperm motility or viability. In addition, they demonstrated that PFAS also did not elevate ROS production or downstream markers of oxidative stress [107]. Nevertheless, PFAS treatment of spermatozoa resulted in a significant retardation of the developmental process of pre-implantation embryos produced in vitro [107,108]. Our results are also in line with those showing that wild boar [109] and human [110] spermatozoa viability is resistant to direct exposure to multiple PFAS molecules. Other studies have reported a reduction in the motility of human spermatozoa exposed to PFOA in vitro, which is likely to be a consequence of changes in membrane fluidity [111]. However, direct comparisons between these studies are confounded by differences in the concentration and composition of the PFAS challenge, as well as the exposure route and duration. In summary, molecular analysis can detect changes that a spermiogram cannot, and these changes could lead to infertility.

These findings have important clinical implications, as they suggest that assessing oxidative parameters and antioxidant capacity could be valuable for early diagnosis of male infertility and monitoring the response to antioxidant treatments. They also indicate that interventions aimed at reducing lipid peroxidation and enhancing antioxidant defences could be beneficial for male reproductive health. This is particularly significant given that research suggests the most reactive lipid aldehydes cause substantial damage at physiological concentrations [92]. Moreover, it is also reported that exposure to increasing concentrations of PFOA of spermatozoa from healthy men with normozoospermia reduced total and progressive sperm motility, impaired chromatin compactness and increased levels of lipid peroxidation and mitochondrial superoxide anion [112]. In addition, PFOS and PFOA altered human sperm capacitation via the cAMP/PKA pathway, increasing sperm ROS and DNA fragmentation during capacitation [113] and one of the most concerning effects of PFOS and PFOA is their association with reduced testosterone levels, similar to clinical observations in infertile men [114]. The main limitation of this study is that it is based on a preliminary analysis of a small sample size. Therefore, analyses of a larger cohort will be performed to confirm these results. The PFAS assessment is limited as it was only conducted in blood serum for PFOA and PFOS, rather than in semen itself, which would have provided new insights into the hypothesised mechanisms of action of PFAS.

## 5. Conclusions

This study is the first to investigate the effects of PFAS exposure on seminal oxidative stress parameters and the ability of SNBPs to protect DNA from oxidative damage. Our findings reveal significant alterations in the redox balance of semen from PFAS-exposed subjects, characterized by elevated LP levels and decreased antioxidant capacity in semen. Particularly concerning was our observation that even the VNT group with grade 0 DNA damage already demonstrated compromised antioxidant capacity and increased LP compared to their VSL group, suggesting that biochemical alterations precede detectable DNA damage. These results emphasise the usefulness of seminal oxidative stress parameters as an early indicator of reproductive health impairment in populations exposed to perfluoroalkyl substances (PFAS). This may be indicative of potential infertility concerns and underscores the necessity for additional investigation into the prolonged consequences of oxidative stress on male fertility and the well-being of offspring.

## Data Availability

All data presented in this paper are available to request corresponding authors.

## Supplementary Materials

The following supporting information can be downloaded at: https://www.mdpi.com/article/10.3390/antiox14070792/s1, Figure S1: Analysis on a 1% agarose gel of pGEM3 plasmid DNA damage induced by H_2_O_2_ in the presence of SNBPs. The different damage grade groups are grade 0 (A), grade 1 (B), grade 2 (C) and grade 3 (D).

## Abbreviations

The following abbreviations are used in this manuscript:

PFAS	PerFluorinated Alkylated Substances
PFOA	Perfluorooctanoic acid
PFOS	Perfluorooctanesulfonic acid
VNT	Veneto region subjects. PFAS contaminated area
VSL	Valley of Sele subjects, Campania region. Not PFAS contaminated area
LP	Lipoperoxides
SNBP	Sperm Nuclear Basic Protein
CuCl_2_	Copper chloride
H_2_O_2_	Hydrogen peroxide
TCA	Trichloroacetic acid
TAC	Total antioxidant capacity
OS	Oxidative stress
ROS	Reactive oxygen species
EIs	Endocrine interfering agents
PMSF	Phenylmethylsulfonyl fluoride
ISDE	International Society of Physicians for the Environment
MRM	Multiple reaction monitoring
ESI-	Electrospray source in negative mode
ARPA	Regional Environmental Protection Agency
