# Supplementary material for: Molecular Alterations in Semen of Per-And Polyfluoroalkyl Substance Exposed Subjects: Association Between DNA Integrity, Antioxidant Capacity and Lipoperoxides
Source: Antioxidants (Basel). 2025 Jun 27;14(7):792. doi: 10.3390/antiox14070792 (PMC12291827; doi:10.3390/antiox14070792)
Supplement: Supplementary file 1 [file antioxidants-14-00792-s001.zip › antioxidants-3655627-supplementary.pdf]

Supplementary Materials

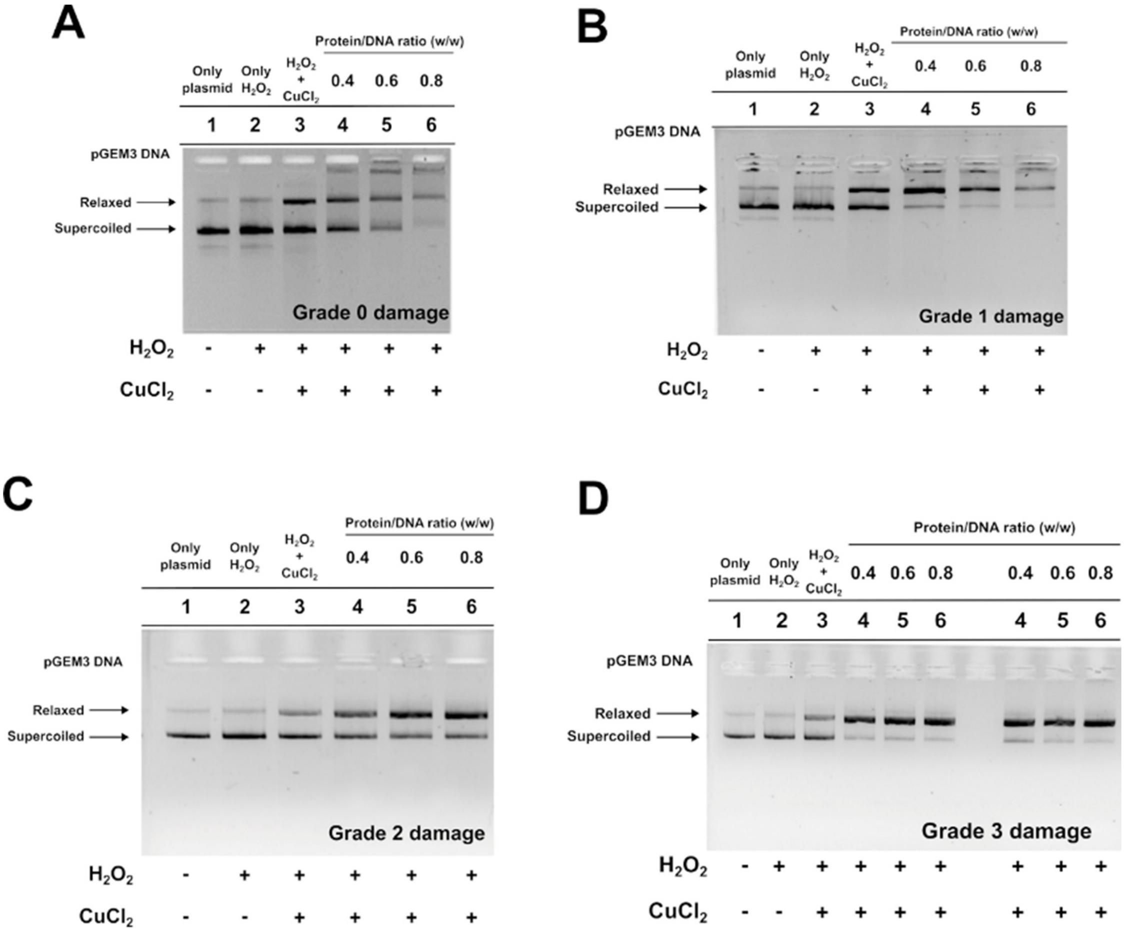

Supplementary Figure 1. Analysis on a 1% agarose gel of pGEM3 plasmid DNA damage induced by H_2O_2 in the presence of SNBPs. The different damage grade groups are grade 0 (A), grade 1 (B), grade 2 (C) and grade 3 (D).
